# Commentary: Ghrelin promotes midbrain neural stem cells differentiation to dopaminergic neurons through the Wnt/β-catenin pathway

**DOI:** 10.3389/fncel.2020.00248

**Published:** 2020-08-21

**Authors:** Jenesis D. Gayden, Zachary Freyberg

**Affiliations:** ^1^Center for Neuroscience, University of Pittsburgh, Pittsburgh, PA, United States; ^2^Department of Psychiatry, University of Pittsburgh, Pittsburgh, PA, United States; ^3^Department of Cell Biology, University of Pittsburgh, Pittsburgh, PA, United States

**Keywords:** neuroregeneration, neuron differentiation, Wnt signaling, neural stem cells, dopaminergic neurogenesis, ghrelin

## Introduction

The regenerative capacity of the adult central nervous system (CNS) has been under intense debate for decades. This process is restricted to specific regions of the brain: the subventricular zone (SVZ) and the subgranular zone (SGZ). To date, the mechanisms responsible for regulating adult neurogenesis remain poorly understood. Several studies have indicated that small molecules like ghrelin play crucial roles in the mechanisms by which neural progenitors turn into functional neurons (Kippin et al., 2005; Berg et al., 2011). Ghrelin, a peptide generated in the stomach, is best known for its roles in appetite regulation. Ghrelin passes through the blood-brain barrier and initiates orexigenic signaling by stimulating growth hormone secretion through activation of hypothalamic receptors (Sovetkina et al., 2020). Recent evidence suggests ghrelin also has key functions in the CNS outside the hypothalamus. Li et al. (2014) showed that exogenous ghrelin treatment promoted proliferation in the SVZ and increased numbers of tyrosine hydroxylase (TH)-expressing dopamine neurons in the olfactory bulb—a key site of neuronal turnover that is innervated by dopamine neurons (Li et al., 2014; Bonzano et al., 2016). Considering these findings, Gong et al. (2020) ask an important mechanistic question: how do neuropeptides like ghrelin affect the process of neural differentiation (Gong et al., 2020)?

Gong et al. (2020) used midbrain neural stem cells (mbNSCs) isolated from the midbrains of rat embryos (E14.5) to investigate the effect of ghrelin on mbNSC proliferation and differentiation. Ghrelin (0.1–1 μM) significantly increased neurosphere diameter as well as expression of TH, the rate-limiting enzyme of DA biosynthesis. This led the authors to conclude that ghrelin promotes dopaminergic neuronal differentiation. Nevertheless, a potential limitation of this conclusion is that the authors did not provide functional validation of the dopaminergic neuronal phenotype beyond demonstrating TH protein expression and measuring total DA content in slices. Indeed, while these cells express TH and produce DA, they may still lack the capacity to efficiently release this *de novo* synthesized DA which would also affect synaptic neurotransmission and connectivity.

Future work can therefore follow-up on the authors' findings by measuring levels of DA release from these cells both basally and in response to stimulation. Furthermore, additional studies can investigate whether these TH-expressing cells can establish the synaptic connections that are observed physiologically in dopaminergic brain regions such as the striatum for effective DA signaling (e.g., activation of DA receptors on spiny projection neurons) (Rice et al., 2011).

To examine the underlying mechanism behind ghrelin's positive effect on dopaminergic neuronal differentiation, Gong and colleagues turned to the Wnt/β-catenin pathway since Wingless-Int (Wnt) proteins have been established as a crucial part of neurogenesis, differentiation, and proliferation (Noelanders and Vleminckx, 2017). Following ghrelin treatment, there was an upregulation of Wnt1 and Wnt3a. Furthermore, DKK1, a Wnt receptor antagonist, blocked the effect of ghrelin on GSK3β phosphorylation and β-catenin. Co-treatment of GSK3β inhibitor + ghrelin produced robust upregulation of phosphorylated GS3Kβ and β-catenin expression, thus highlighting ghrelin's regulatory effect on this pathway. Overall, these findings show that dopaminergic neuronal differentiation is synergistically mediated by ghrelin and the Wnt/β-catenin pathways.

## Discussion

Though the mechanisms behind the interplay between Wnt/β-catenin, DA, and ghrelin signaling remain poorly understood in brain, other organ systems may yield new clues. Han et al. (2019) reported in kidney that decreased dopamine D_2_ receptor (D2R) activation leads to increased renal cell proliferation, mediated by Wnt3a. Furthermore, Han et al. (2019) showed that D2R-mediated phospho-AKT inactivation increases levels of non-phosphorylated GSK3β, thus leading to more degradation of β-catenin (Han et al., 2019). This work brings forth the hypothesis that G protein-coupled receptors (GPCRs) such as D2R may modulate Wnt/β-catenin signaling. The ghrelin receptor is also a GPCR which suggests a broader phenomenon in which GPCR-dependent activity acts as a catalyst and/or modulates neuronal differentiation via the Wnt/β-catenin pathway.

**Figure 1:**
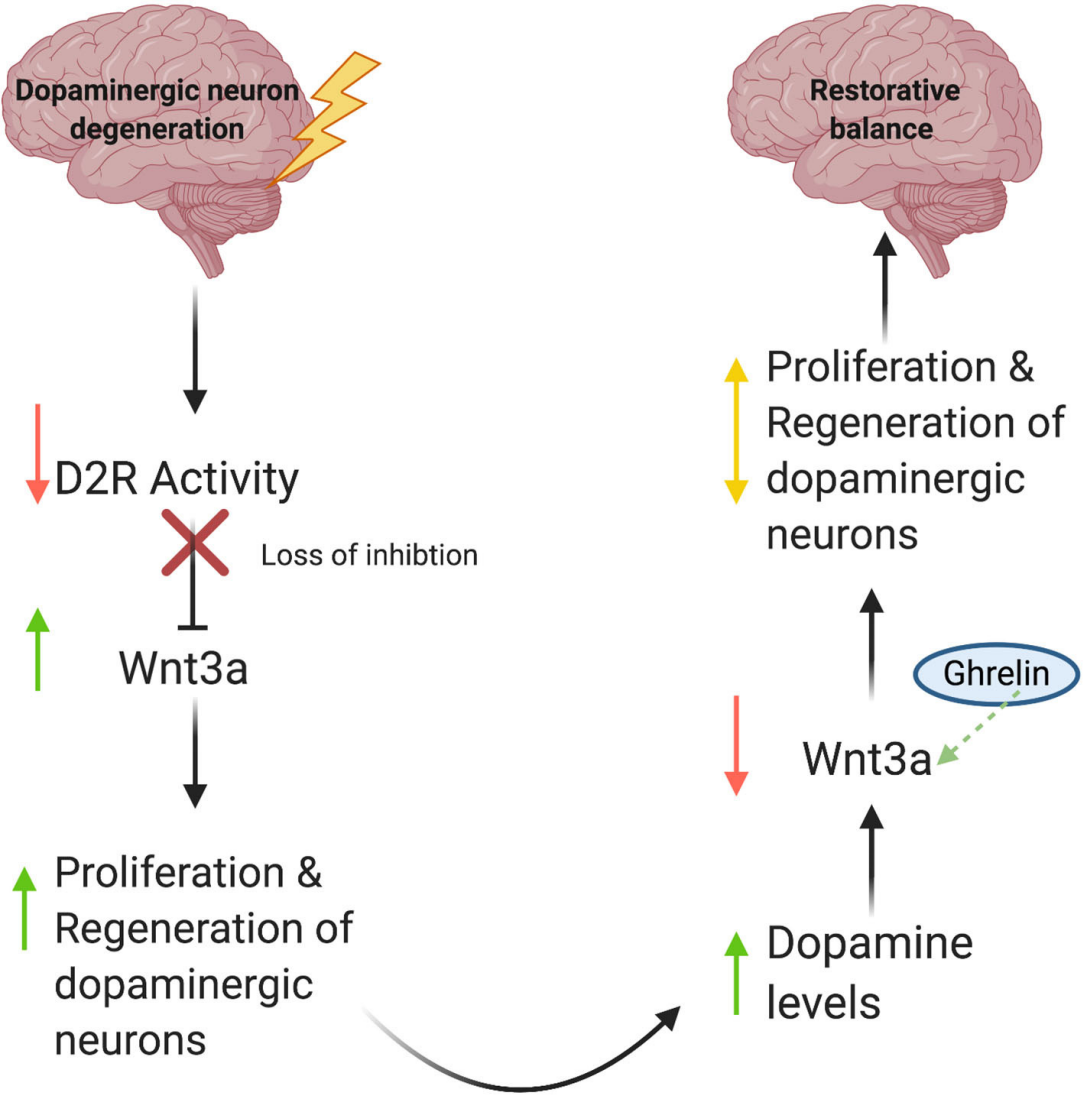
A model for Wnt-mediated neuronal regeneration. Degenerative disease processes lead to dopaminergic neuron loss, resulting in decreased synaptic D2R activity. This decrease in activity releases the inhibitory brake on Wnt3a production and increases Wnt3a levels, consequently stimulating neural stem cell proliferation and restoring cellular and synaptic dopamine levels. As DA levels rise, inhibitory D2R activity increases, which then diminishes levels of Wnt3a. Ghrelin compensates for this decrease by serving as a critical counterbalance and increasing Wnt3a expression in response. As a result, proliferation and regeneration of dopaminergic neurons is auto-regulated, thus preventing unchecked cell proliferation.

Gong et al. (2020) report increased mRNA levels of Wnt3a and Wnt1, as well as activation of the Wnt/β-catenin pathway in the ghrelin-treated groups, suggesting a role for ghrelin in adult neurogenesis. Based on these data, we propose the following model (Figure 1): (1) Degenerative DA neuron loss results in a decrease in synaptic D2R activity; (2) Because D2R is an inhibitory modulator of Wnt3a synthesis (Han et al., 2019), this leads to increased production of Wnt3a and possibly Wnt1; (3) Increased Wnt1/3a signaling then stimulates cell proliferation of neural stem cells and subsequent regeneration of dopaminergic neurons, albeit in a limited manner. Conversely, during periods of increased D2R activity, we would expect decreases in Wnt3a expression as part of a regulatory feedback loop to prevent extended periods of uncontrolled cell proliferation (Han et al., 2019). Since ghrelin increases Wnt3a expression, it may function as a critical counterbalance to dopaminergic signaling by mitigating loss of Wnt3a expression and signaling brought on by increases in D2R activity. Ultimately, we propose that the recent discovery of an autoregulatory promotor element within the *Wnt3a* gene functions to keep *de novo* Wnt3a synthesis in check and prevents uncontrolled cell proliferation to maintain appropriate levels of neurogenesis (Han et al., 2019).

Regeneration of DA neurons holds immense therapeutic potential for pathologies associated with DA neurodegeneration, most notably in Parkinson's Disease (PD). Differentiation of mature midbrain DA neurons from embryonic stem cells has long been a goal of potential therapies (Kim et al., 2002), with studies showing that such an approach can partially mitigate behavioral deficits stemming from DA neuron loss in different animal models as reviewed by Song et al. (2018). Despite modest success, embryonic stem cell-based therapies pose potential problems such as the possibility of inducing development of teratomas of multiple unwanted cell types. In contrast, neural stem cells (NSCs) offer a more advantageous therapeutic strategy over embryonic stem cells since NSCs are already restricted to a certain cell type. Moreover, an NSC-based strategy would therefore eliminate the threat of teratoma development, as well as potentially diminish the risk of patient immune rejection (Gale and Li, 2008). Nevertheless, NSCs also pose their own problems. While studies have confirmed that cells derived from fetal midbrain can help therapeutically in PD animal models, these cells are limited as a source of dopaminergic neurons because their ability to generate these neurons is unstable (Kim et al., 2002). The use of ghrelin to promote production of fully differentiated dopamine neurons may help to address the challenge of providing an effective method for producing dopaminergic neurons. However, the long-term survival of these cells *in vivo* in an adult animal model remains in question.

Overall, Gong et al. (2020) demonstrate ghrelin's positive effect on dopaminergic neuronal differentiation from NSCs via Wnt/β-catenin signaling. However, much work must be done to establish NSCs as a viable method of neuronal regeneration. Ultimately, elucidating ghrelin's effects on dopaminergic neurons offers the promise of a novel therapeutic solution to neurodegenerative diseases of the DA system.

## Author Contributions

JDG prepared the figure. JDG and ZF drafted the manuscript, edited and revised the manuscript, and approved the final version of manuscript. Figure created with Biorender.com. All authors contributed to the article and approved the submitted version.

## Conflict of Interest

The authors declare that the research was conducted in the absence of any commercial or financial relationships that could be construed as a potential conflict of interest.
